# Supplementary material for: Thraustochytrium sp. and Aurantiochytrium sp.: Sustainable Alternatives for Squalene Production
Source: Mar Drugs. 2025 Mar 19;23(3):132. doi: 10.3390/md23030132 (PMC11944157; doi:10.3390/md23030132)
Supplement: Supplementary file 1 [file marinedrugs-23-00132-s001.zip › marinedrugs-3443191-supplementary.pdf]

Supplementary material

Thraustochytrium sp. and *Aurantiochytrium* sp.: Sustainable Alternatives for Squalene Production

Júnior Mendes Furlan [1,*], Graciela Salete Centenaro [1], Mariane Bittencourt Fagundes [2,*], Carlos Borges Filho [1], Irineu Batista [3] and Narcisa Bandarra [3]

[1] Chromatography and Food Analysis Research Group, Federal University of Pampa, Itaqui 97650-000, RS, Brazil

[2] Interdisciplinary Centre of Marine and Environmental Research CIIMAR, 4450-208 Matosinhos, Porto, Portugal

[3] Portuguese Institute for Sea and Atmosphere, 1495-006 Lisbon, Portugal

* Correspondence: juniorfurlan@unipampa.edu.br (J.M.F.); mfagundes@ciimar.up.pt (M.B.F.)

Table S1. Economic Parameters and Cost Breakdown:

Parameter / Category	Value / Cost (USD)
CAPEX (Investment Cost)	$50 million USD
OPEX (Annual Operating Cost)	$10 million USD
Discount Rate	8% per year
Project Lifespan	20 years
Production per Reactor	59,531.5 kg/year
Market Price of Squalene	$100/kg
Total Annual Production	2,500 metric tons/year
Raw Materials - Glucose	$29,750
Raw Materials - Ammonium Sulfate	$11,900
Raw Materials - Yeast Extract	$892,500
Energy Consumption	$50,000
Labor	$100,000
Maintenance	$30,000
Total OPEX	$480,288
